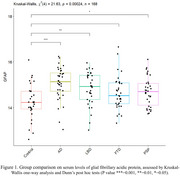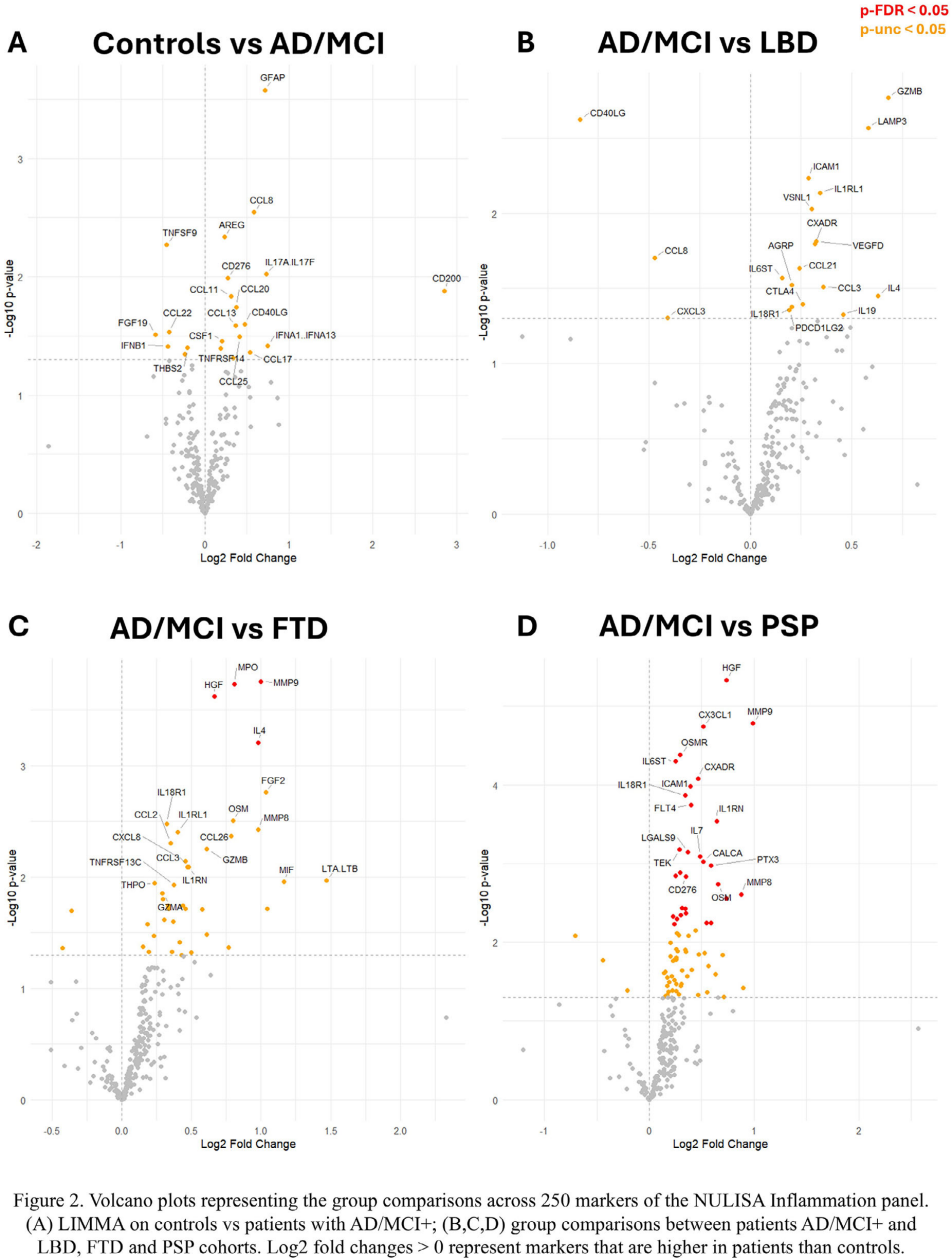# Blood‐derived patterns of inflammation across neurodegenerative diseases

**DOI:** 10.1002/alz70856_102670

**Published:** 2025-12-25

**Authors:** Robert Durcan, Amanda J Heslegrave, Peter Swann, Julia Goddard, Leonidas Chouliaras, Henrik Zetterberg, James B Rowe, John T O'Brien, Maura Malpetti

**Affiliations:** ^1^ University of Cambridge, Cambridge, Cambridgeshire, United Kingdom; ^2^ UK Dementia Research Institute at UCL, London, United Kingdom; ^3^ University of Cambridge, Cambridge, United Kingdom; ^4^ University of Cambridge, Cambridge, ‐, United Kingdom

## Abstract

**Background:**

Neuroinflammation is involved in the pathophysiology of several neurodegenerative diseases and has been linked to faster clinical decline. This study evaluates a novel multiplex proteomic method to assess blood‐based inflammation patterns in patients with neurodegenerative diseases, including Alzheimer's disease (AD), Lewy body dementia (LBD), frontotemporal dementia (FTD) and progressive supranuclear palsy (PSP).

**Method:**

Serum samples from *n* = 137 patients (AD/MCI+=36, LBD=33, FTD=35, PSP=33) and *n* = 29 age‐matched controls were analysed with the NUcleic acid Linked Immunosorbent Assay (NULISA) Inflammation 250 panel. The panel measures ∼250 analytes, including Glial fibrillary acidic protein (GFAP) and other biomarkers of inflammation and immune response. GFAP levels were comapared across groups with a Kruskal‐Wallis test. All 250 biomarkers were compared across groups with Linear Models for Microarray and RNA‐Seq Data Analyses (LIMMA), correcting for age and sex.

**Result:**

Serum levels of GFAP were increased especially in patients with AD/MCI+ and LBD and to a lower extent in patients with PSP (Figure 1). Considering all 250 markers, patients with AD/MCI+ showed increased GFAP levels as compared to controls over and above other markers (Figure 2A). Comparing each patient group to the AD/MCI+ cohort, patients with LBD had a larger number of markers being upregulated than AD/MCI+ (Figure 2B), including Granzyme Β (GZMB) and Lysosome‐associated membrane glycoprotein 3 (LAMP‐3). Patients with FTD (Figure 2C) and PSP (Figure 2D) had higher levels in several inflammation markers as compared to AD/MCI+, including Matrix metalloproteinase‐9 (MMP9) and Hepatocyte Growth Factor (HGF).

**Conclusion:**

The NULISA Inflammation 250 panel demonstrates high sensitivity for detecting inflammatory patterns across neurodegenerative disorders. It revealed distinct condition‐specific profiles. Patients with LBD, FTD and PSP showed upregulation of many inflammation markers, as compared to controls and patients with AD.